# Comprehensive Rehabilitation of a Patient With Foot Drop Secondary to Lumbar Canal Stenosis: A Case Report

**DOI:** 10.7759/cureus.52275

**Published:** 2024-01-14

**Authors:** Mahek R Mohani, Neha Arya, Grisha Ratnani, Pallavi Harjpal, Pratik Phansopkar

**Affiliations:** 1 Neurophysiotherapy, Ravi Nair Physiotherapy College, Datta Meghe Institute of Higher Education and Research, Wardha, IND; 2 Neurophysiotherapy, Ravi Nair Physiotherapy College, Datta Meghe Institue of Higher Education and Research, Wardha, IND

**Keywords:** case report, physiotherapy, physiotherapy rehabilitation, spinal fusion, lumbar decompression, foot drop, lumbar canal stenosis

## Abstract

One of the most prevalent degenerative musculoskeletal conditions is lumbar spinal canal stenosis (LSS), which is characterized by narrowing of the lumbar spinal canal that pressures the nerve roots and cauda equine. LSS, when treated surgically, usually presents with foot drop as its major complication. Foot drop is a common presentation of several clinical diseases, traditionally characterized as severe weakening of ankle and toe dorsiflexion. Foot drop has a great impact on patients' lives, lowering their quality of life and affecting their activities of daily living. Ankle dorsiflexion weakness leads to foot drop and a high-stepping gait, which can cause multiple falls and accidents. This case study aimed to assess the efficacy of a customized physiotherapy program in a 50-year-old woman with paraparesis along with left foot drop and post-surgery complications following lumbar decompression and spinal fusion at L3-S1 (lumbar-sacral) level after a jerk experienced by her while working out in the gym. The objective was to determine the impact of individualized exercises on the patient's strength, gait, balance, and pelvic floor function over a 12-week rehabilitation period. The interventions included lower limb exercises (stretching exercises, strengthening exercises, and weight-bearing exercises), pelvic floor exercises, and core stability training. The findings demonstrated significant improvements in the patient's functional outcomes, as evidenced by enhanced scores in the Berg Balance Scale, Manual Muscle Testing, Dynamic Gait Index, Barthel Index, and Stanmore Assessment Questionnaire. Notable progress was observed in the strength, balance, gait, and pelvic floor function, highlighting the positive influence of targeted physiotherapeutic interventions. This case underscores the importance of tailored exercise plans in addressing the complexities of post-surgery challenges, emphasizing the potential for comprehensive recovery and improved overall quality of life through personalized physiotherapy.

## Introduction

Degenerative lumbar spinal stenosis (LSS) is the narrowing of the spinal canal as a result of degenerative changes in the ligamentum flavum, intervertebral discs, and spinal joints. Spinal canal stenosis (SCS) is an incorrect decrease of the spinal route or neighboring recesses. The region around the neurovascular tissue may constrict, leading to major clinical symptoms such as neurological claudication, radiating pain in the lower limbs, lower back discomfort, and difficulty urinating and defecating. Medication, physical therapy, and possibly surgery are common treatment options [1]. Using radiological diagnosis to assess the presence of LSS, the pooled prevalence is 11% in the asymptomatic population, 38% in the general population, 15% in primary care patients, 32% in secondary care patients, and 21% in the general population from primary and secondary care [2]. Foot drop is a unique symptom of a serious motor impairment brought on by degenerative lumbar disorders. The two main lumbar disorders associated with foot drop are spinal stenosis and disc herniation. Although it is also commonly identified at the L5/S1 level and several levels, the disease is typically found at the L4/5 spinal level. An electrodiagnostic evaluation is essential for distinguishing foot drop from peripheral and spinal causes. Ankle dorsiflexion weakness leads to foot drop and high-stepping gait, which can cause multiple falls and accidents [3]. The most common reasons are compression of the peroneal nerve and injury to the spinal lumbar nerve root. Foot drop is a complex and multifaceted ailment; in 18% of cases, the presence of numerous medical disorders is found to be a potential contributing factor [4].

Congenital, degenerative, and other causes are the three broad groups into which the etiology of LSS falls. LSS is a degenerative condition that is commonly observed in middle-aged and elderly individuals [5]. The word "foot drop" typically describes a marked weakening in the dorsiflexion of the ankle and toe [3]. Currently, available treatment options for mild impairments include surgery and functional electrical stimulation (FES) devices. Therapy alternatives include fixed ankle-foot orthoses (AFO) if the weakness is more severe [6]. By reducing pain and restoring muscle strength and range of motion, early physical therapy intervention speeds up patients' clinical recovery [7]. A foot drop occurs when the front portion of the foot is immobile. It is linked to both disc herniation and spinal canal stenosis, two prevalent degenerative lumbar diseases. A detailed analysis of 46 cases of foot drop associated with lumbar disease reveals that 35% of the cases are attributable to spinal stenosis and 52% to disc herniation [8]. Foot drop is characterized by pronounced weakness in the dorsiflexion of the ankle and toe [9]. Ankle-foot orthoses (AFOs) are commonly utilized in mechanical foot drop therapies to preserve the dorsiflexion of the foot. Function, stabilizing gait, distributing the load to manage pain, and delaying the development of fixed deformities are all aided by an externally attached device to the limb, like an ankle-foot orthosis (AFO) [10]. Various studies have shown significant improvement in multiple aspects like strength, gait, balance, and pelvic floor function and these findings emphasized the role of physiotherapy in optimizing the recovery outcomes for individuals with complex neurological issues. As a result, a customized physical therapy program was designed with the goals of reducing tendonitis tightness, strengthening the pelvic floor muscles, preventing muscle atrophy due to denervation, improving balance and gait, and improving daily living activities (ADL).

## Case presentation

Patient information

A 50-year-old female presented with chief complaints of walking difficulties, challenges in performing toileting activities, and bowel and bladder incontinence persisting for three months. Additionally, she reported weakness in both lower limbs and an inability to lift her left foot for the past month. The patient was apparently alright six years ago when she suddenly experienced a jerk on her back while working out in the gym, after which she started having lower back pain. Back pain was intermittent and was sharp shooting type, which aggravated on movement and relieved on rest. The patient visited a local practitioner, and she was diagnosed with a lumbar strain; medications were prescribed; however, the pain did not alleviate. The patient also has a history of hypertension for six years. For the past two months, symptoms aggravated, for which she visited our hospital, and an MRI (Magnetic resonance imaging) was done, which showed stenosis at the level of L3-S1 and was recommended for surgery. She was operated on for posterior decompression and spinal fusion at the L3-S1 (lumbar-sacral) level in early September 2023, after which she was discharged. Her back pain was relieved, but the bowel and bladder incontinence with weakness in both the lower limbs persisted. The patient is diagnosed with paraparesis and foot drop of the left foot, with the following complaints; now, the patient is admitted to the orthopedic ward and is advised for physiotherapy.

Clinical findings 

Before the commencement of the examination, the patient gave her informed consent and underwent the examination. The patient was conscious and cooperative. On neurological examination, the range of motion (ROM) of the affected extremity was taken before and after the rehabilitation. The manual muscle testing (strength) assessment was done before rehabilitation, according to Oxford grading. The tone in both the upper extremities and left lower extremity was found to be +2 (normal), but the tone in the right lower limb was found to be +1 (hypotonia), according to the tone grading scale. According to the American Spinal Injury Association (ASIA) impairment scale, the grade of injury is D, motor incomplete, the motor score for the upper extremity was 50, the lower limb was 38, and the motor level was L3. The sensory level was S1, and score for pinprick sensation was 96, and the light touch was 96. Table 1 depicts an examination of deep tendon reflexes. Table 2 shows the range of motion examination pre-rehabilitation.

**Table 1:** Assessment of deep tendon reflexes of left lower limb + Diminished reflex

Reflexes	Right	Left
Knee	+	+
Ankle	+	Absent
Plantar reflex	flexor	flexor

**Table 2:** ROM examination pre-rehabilitation ROM - range of Motion; AROM - active range of motion; PROM - passive range of motion; N/A - not assessable

Joint	Pre-rehabilitation			
	Left		Right	
	AROM	PROM	AROM	PROM
Hip flexion	0-35^0^	0-60^0^	0-40^0^	0-65^0^
Hip extension	0-5^0^	0-10^0^	0-5^0^	0-10^0^
Hip abduction	0-10^0^	0-35^0^	0-10^0^	0-35^0^
Hip adduction	0-10^0^	0-12^0^	0-12^0^	0-15^0^
Internal rotation	0-15^0^	0-20^0^	0-15^0^	0-20^0^
External rotation	0-20^0^	0-28^0^	0-25^0^	0-35^0^
Knee Flexion	0-35^0^	0-130^0^	0-40^0^	0-130^0^
Ankle Dorsiflexion	Not able to perform	0-10^0^	0-10^0^	0-15^0^
Ankle Plantarflexion	N/A	0-30^0^	0-28^0^	0-35^0^

**Figure 1:**
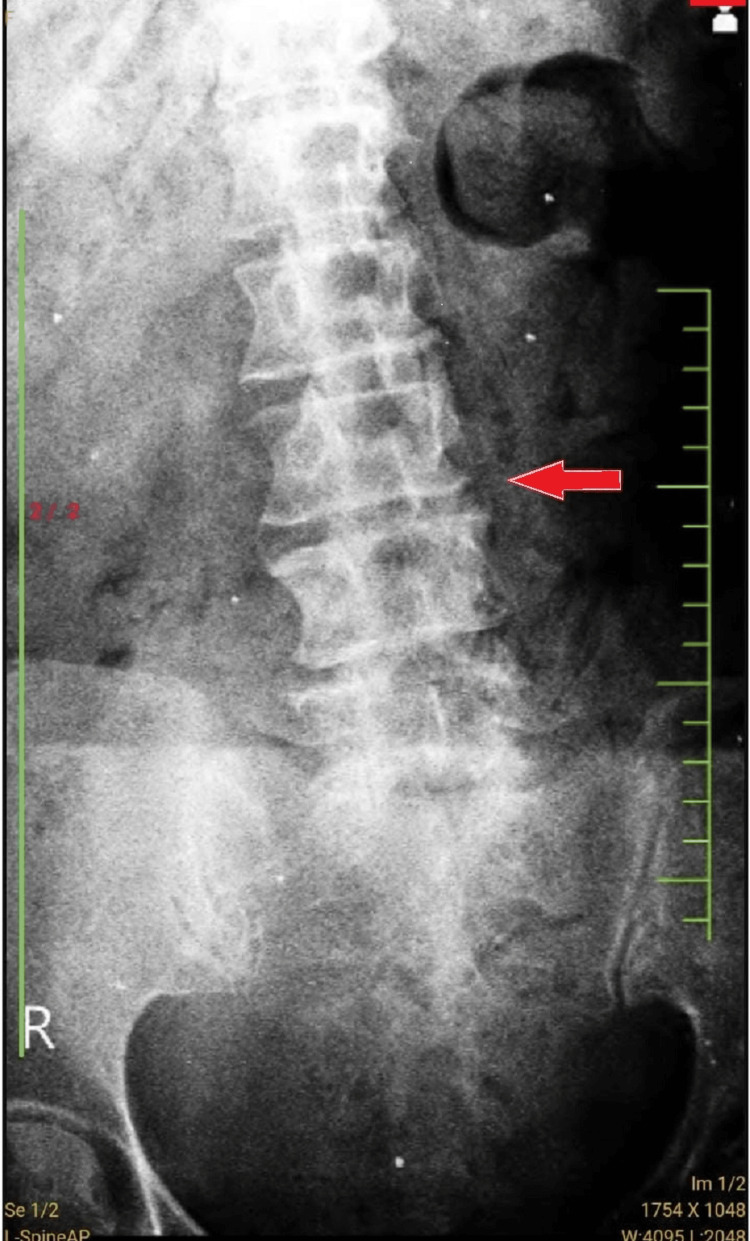
Shows the X-ray of lumbar compression at level L3 to S1 level (red arrow) L - lumbar, S - sacral

**Figure 2:**
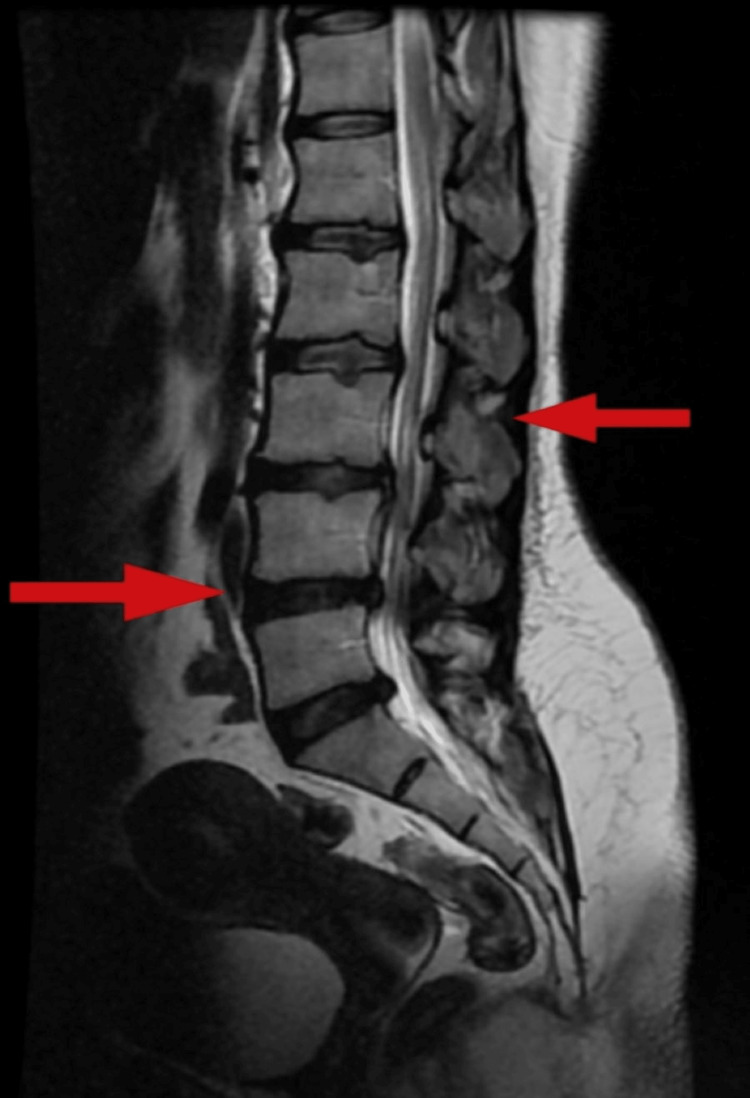
Pre-operative MRI of the spine showing stenosis at level of L3-S1 (red arrow) MRI - magnetic resonance imaging

The X-ray revealed the presence of lumbar compression at level L3-S1, which is depicted in Figure 1, and Figure 2 illustrates an MRI spine showing stenosis at level L3-S1.

**Figure 3:**
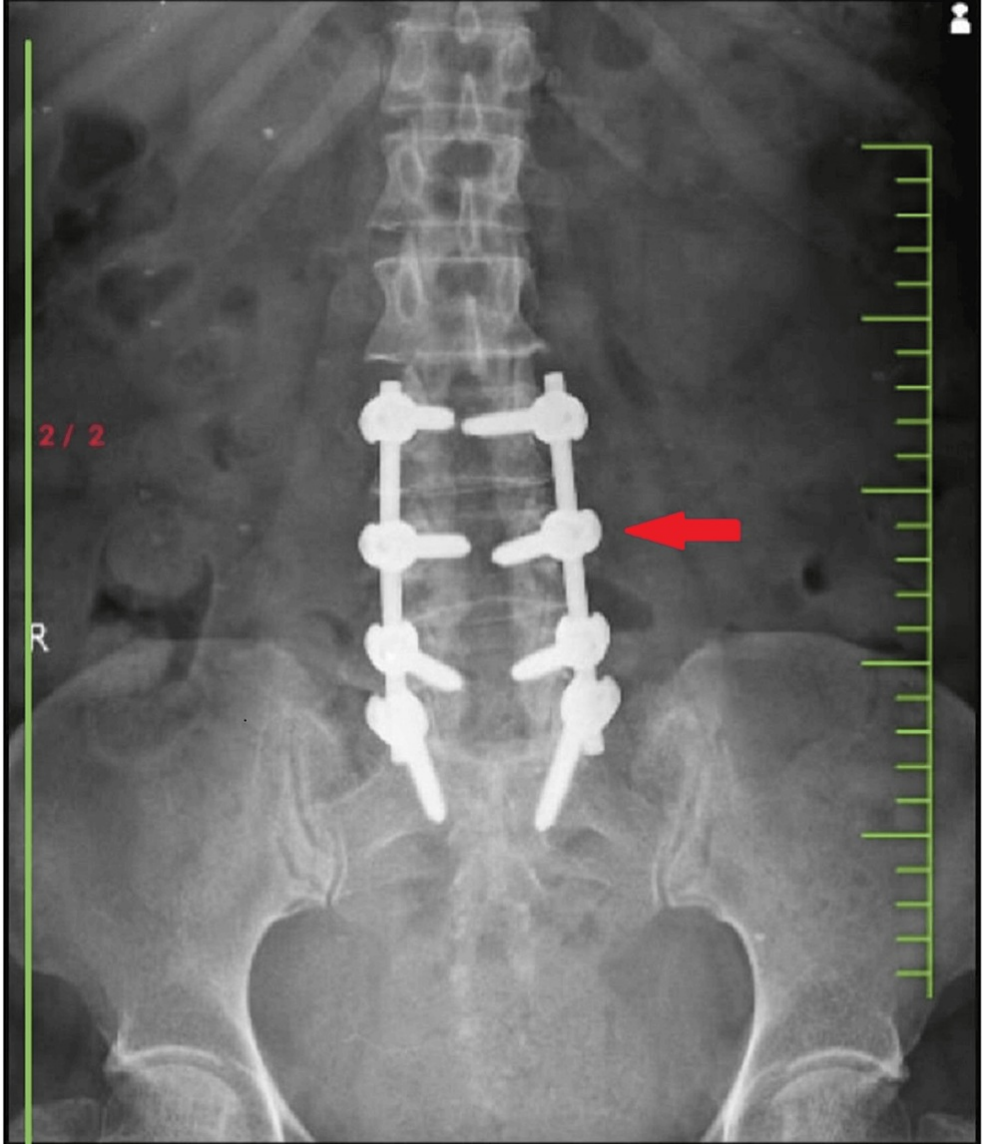
Post-operative CT scan of the lumbar spine after lumbar decompression and spinal fusion at level L3 to S1 using Pedicel screw (red arrow) CT - computed tomography

A CT scan shows the use of pedicel screws for fixing after lumbar decompression and spinal fusion surgery at levels L3-S1, which is depicted in Figure 3.

Physiotherapy intervention

A customized rehabilitation plan was made for the patient. The patient was given physiotherapy rehabilitation for 12 weeks; sessions were scheduled for five days each week for nearly 40 minutes every day. The physical therapy rehabilitation plan describing the goals, intervention, and regimen is shown in Table 3 below.

**Table 3:** Rehabilitation protocol TA - tendoachilles; ADL - activities of daily living; PRT - progressive resistance training; SLR - straight leg raise

Goals	Intervention	Regimen
Patient education	Explaining the relatives of the patient the advantages of physical therapy. To inform patients about different positions that could be helpful to them.	The impact of physical activity, walking, and posture were discussed with the patient and caregivers during counseling. Education regarding pillow positioning was provided to the patient's caregiver.
Prevention of denervation atrophy	By electrical stimulation between 30 and 50 Hz in frequency, the lateral and anterior compartments were stimulated.	Twice a day, for one set of ten repetitions. Progression from 2 weeks. 15 repetitions in one set, twice a day. Week 3 and 4: Do two sets of 20 repetitions each.
To treat bowel and bladder incontinence by increasing pelvic floor muscle strength	Kegel's exercise, pelvic bridging, static back exercises, core strengthening, and breathing exercises.	One set of 10 repetitions
To reduce joint stiffness, enhance range of motion, and preserve thigh muscle strength.	Using the PRT for lower limb (hip flexors, hip extensors, abductors, and adductors).	10 repetitions X 1 set twice a day for 2 weeks
		Increase to 20 repetitions in 1 set thrice a day in week 3 to week 4
		30 repetitions in 1 set in week 4 to week 5
		Heel slides are performed in one set X 10 repetitions
To reduce Tendoachilis (TA) tightness	By stretching the TA, dorsiflexion will remain flexible as usual. Advanced in week 4 and kept going until week 12.	30 seconds hold with 3 sets
Facilitate weight bearing	Combining pivot shifting with a partial weight bearing. Between weeks 3 and 4, the patient transitioned from prolonged sitting to upright sitting, then standing re-education, and finally walking re-education. In week 6, the re-education of standing and walking advanced.	Initiated after the 3rdweek
To improve the strength of foot muscles	Intrinsic exercise for foot. Active Assisted Range of motion exercise for the foot.	One set X 10 repetitions
Focusing on ADLs	During week four, taking care of oneself, dressing, and using the western toilet. ADLs become independent between weeks 9 and 12.	Starting from week 4
Gait training	It was encouraged to walk in tandems, ascend stairs, move ahead and backward, step climb, and navigate obstacles.	Starting from week 5

**Figure 4:**
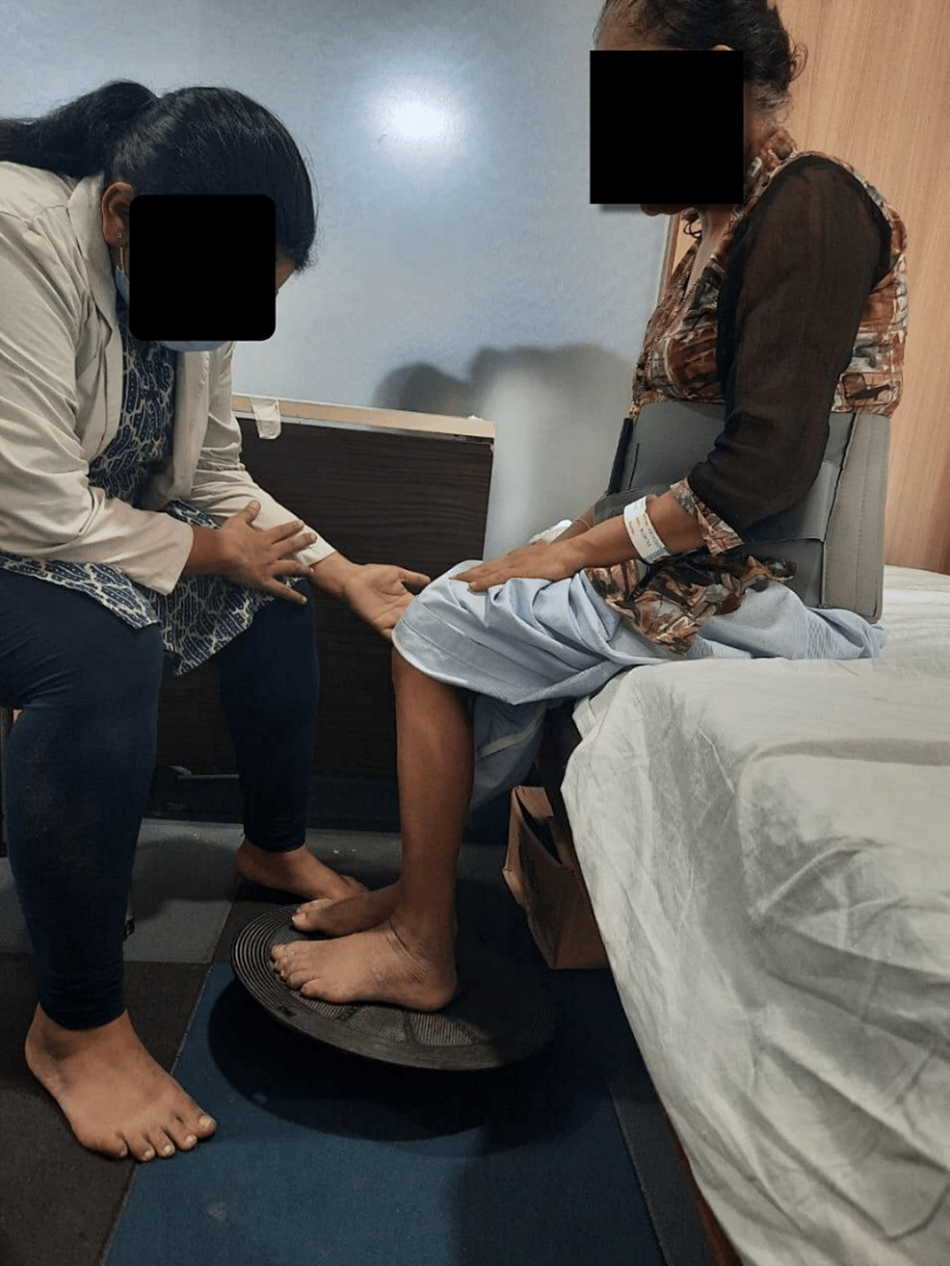
Patient performing wobble board activity in sitting

**Figure 5:**
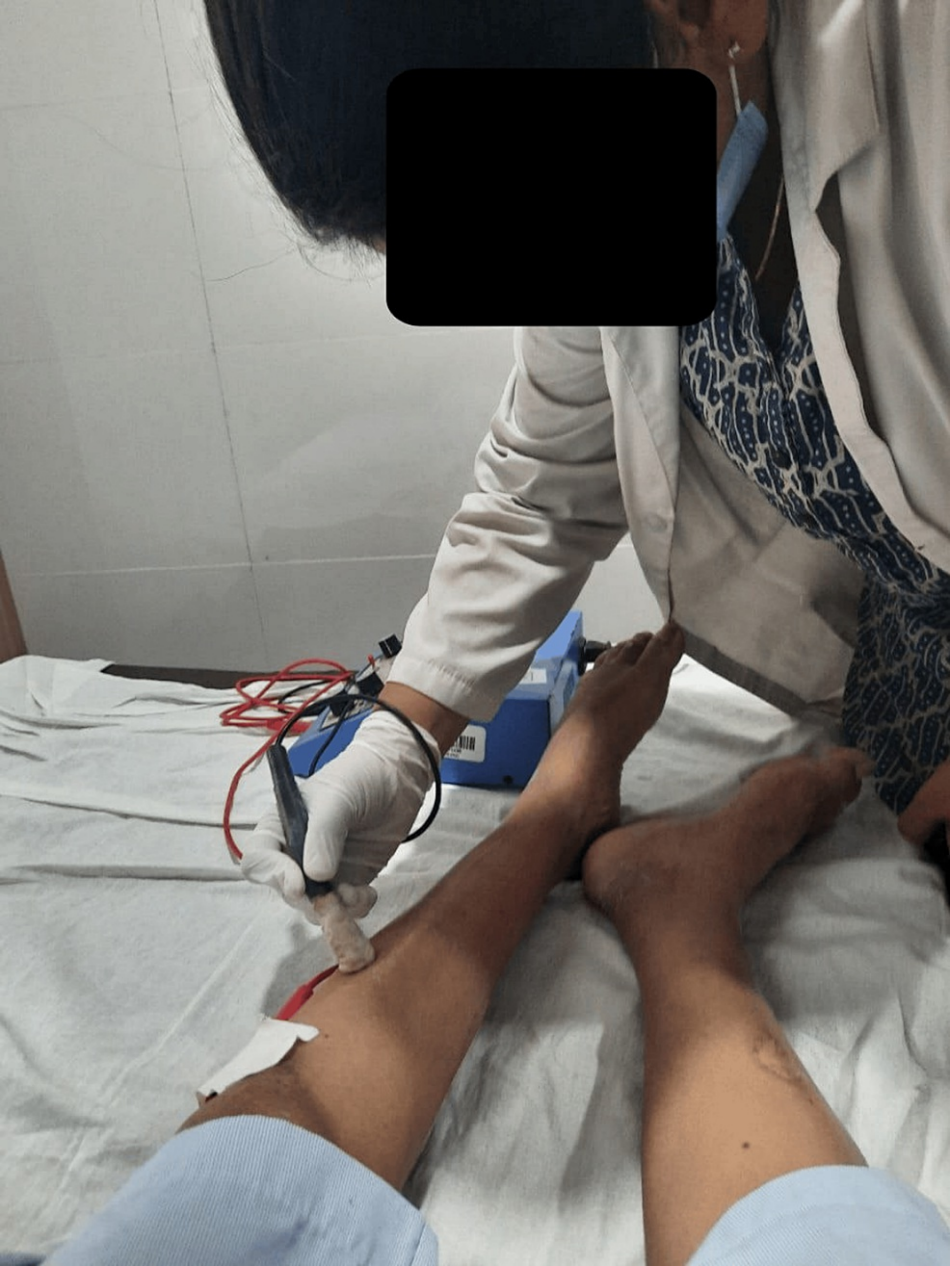
Patient being treated with electrical muscle stimulation

**Figure 6:**
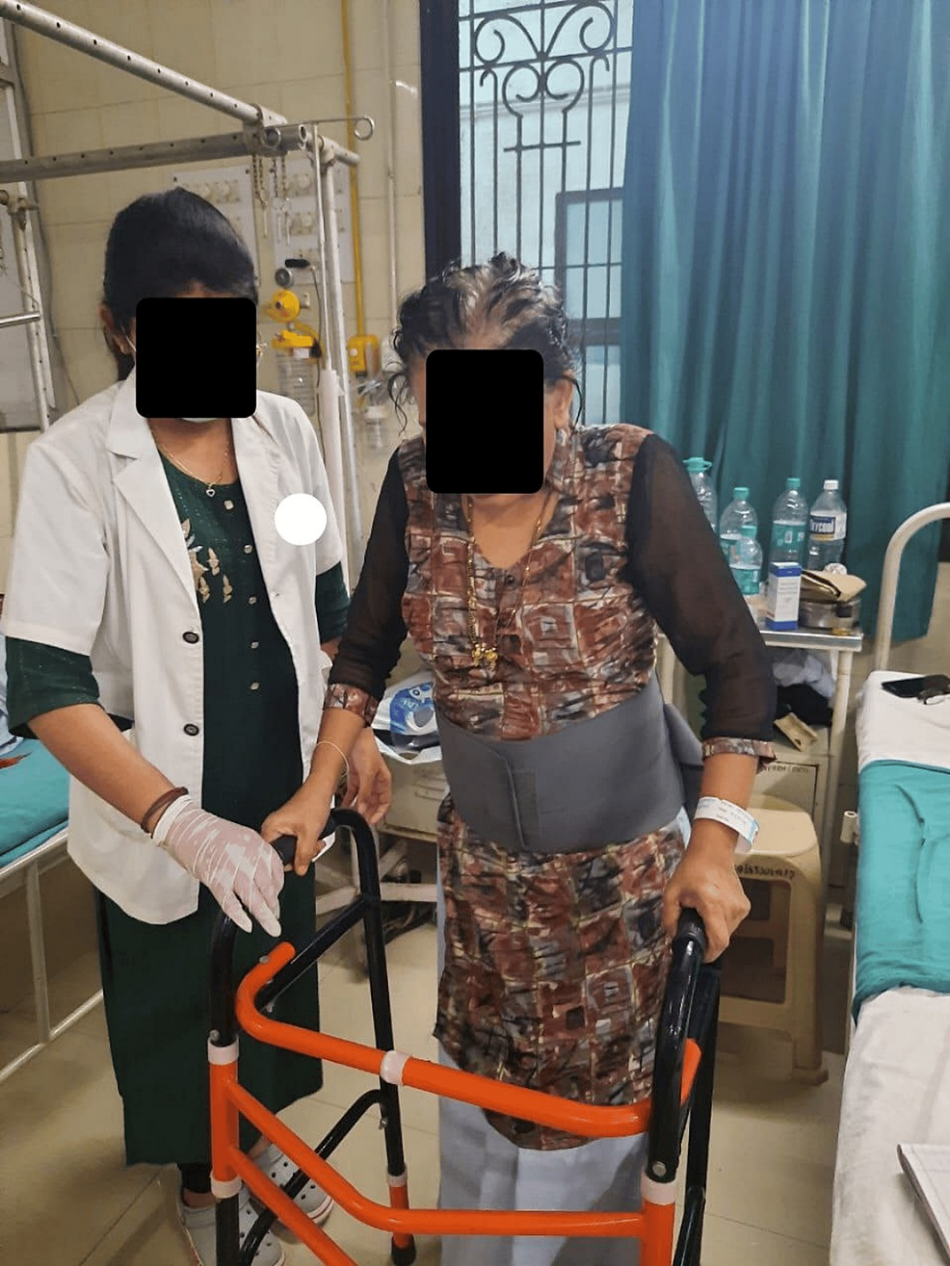
Patient performing ambulation with the support of a walker

 Figures 4, 5, 6 depict the patient performing various physiotherapy exercises.

Outcome measures

The patient was assessed using the Berg Balance Test, Dynamic Gait Index, Barthel Index, Stanmore Assessment Questionnaire, and Manual Muscle Testing, and the scores were recorded pre-rehabilitation and post-rehabilitation. The PERFECT (P- power, E- endurance, R- repetitions, F- fast contractions, ECT- every contraction timed) scheme is a method of examination of the pelvic floor muscles. It was used to evaluate the strength of pelvic floor muscles pre-rehabilitation; the power was grade 2, and the contraction was held for three seconds and repeated three times (with three seconds rest between each contraction). The patient was unable to perform fast contractions. Post-rehabilitation power was grade 4, and contraction was held for six seconds, repeated five times, followed by nine fast contractions (power grading, 1- flicker, 2- weak, 3- moderate, 4- good, 5- strong). Table 4 depicts ROM examination post-rehabilitation.

**Table 4:** Post-rehabilitation ROM examination ROM - range of motion

Joint	Post-rehabilitation			
	Left		Right	
	AROM	PROM	AROM	PROM
Hip flexion	0-65^0^	0-90^0^	0-70^0^	0-90^0^
Hip extension	0-20^0^	0-25^0^	0-20^0^	0-30^0^
Hip abduction	0-30^0^	0-35^0^	0-35^0^	0-40^0^
Hip adduction	0-25^0^	0-30^0^	0-30^0^	0-30^0^
Internal rotation	0-25^0^	0-35^0^	0-20^0^	0-30^0^
External rotation	0- 25^0^	0-30^0^	0-30^0^	0-35^0^
Knee flexion	0-90^0^	0-130^0^	0-100^0^	0-130^0^
Ankle dorsiflexion	0-8^0^	0-15^0^	0-18^0^	0-20^0^
Ankle plantarflexion	0-10^0^	0-35^0^	0-35^0^	0-40^0^

Table 5 shows assessment findings for outcome measures.

**Table 5:** Outcome measures

Outcome Measures	Pre-rehabilitation	Post-rehabilitation
Berg Balance Scale	27/56	35/56
Dynamic Gait Index	10/24	15/24
Barthel Index	65/100	70/100
Stanmore Assessment Questionnaire	50/100	55/100

Table 6 shows findings for Manual Muscle Testing (MMT).

**Table 6:** Manual Muscle Testing (strength) assessment according to Oxford gradings 5+: Complete ROM against gravity with maximal resistance, 4: complete ROM against gravity with moderate resistance, 3+: complete ROM against gravity with minimal resistance, 3: complete ROM against gravity, 3-: some but not complete ROM against gravity, 2+: Initiates motion against gravity, 2: complete ROM with gravity eliminated, 2-: Initiates motion if gravity is eliminated, 1: Evidence of slight contractility but no joint motion, 0: No contraction palpated ROM - range of motion

Muscles	Pre-rehabilitation		Post-rehabilitation	
	Left	Right	Left	Right
Hip flexors	2+	2+	3-	3-
Hip extensors	2-	2-	3-	3-
Knee flexors	2+	2+	3	3
Knee extensors	2+	2+	3-	3-
Ankle dorsiflexors	1	3-	2-	3
Ankle plantar flexors	N/A	2+	2+	3-

## Discussion

The case mentioned above is of a 50-year-old female diagnosed with paraparesis along with left foot drop and bowel incontinence and involuntary urination post lumbar decompression and spinal fusion at the L3-S1 level. For this patient, a tailored physiotherapy program was planned. Different outcome measures, like the Dynamic Gait index, Berg Balance Scale, etc., were used before and after the patient was given a customized physiotherapy program. Findings showed significant improvement in various aspects like strength, gait, balance, range of motion, and pelvic floor function. The findings emphasized the role of physiotherapy in optimizing the recovery outcomes for individuals with complex neurological issues.

A foot drop results in an improper walking form and a fall. Step-down stimuli have an orthodontic impact right away, as seen in the functional electrical stimulation (FES) - induced ankle dorsiflexion, which improves the biomechanics of gait [11]. Jaqueline da Cunha et al. conducted a study in which she found that FES in conjunction with supervised exercises (such as physiotherapy) was better compared to overseen exercises alone in enhancing gait speed. Ankle dorsiflexion, balance, and functional mobility are all improved with FES [12]. Ankle-foot orthoses (AFO) are frequently prescribed to treat foot drop. Another method for correcting foot drop is functional electrical stimulation (FES). Unlike AFOs, FES increases the force-generating capacity of muscles, encourages active contractions of those muscles, helps prevent disuse atrophy, decreases muscle tone and spasms, makes the use of proximal limb muscles more energy-efficient, and facilitates motor relearning [13]. Muscle force production can be increased with progressive resistance exercise (PRE). PRE increases force production, which further increases the capacity for carrying out daily tasks [14]. Weight-bearing exercises can help restore the ankle and leg to their original strength [15]. To reduce tension on the fused or neighboring segment, strengthening activities should be done in a neutral lumbar position in the early postoperative period [16].

In order to promote core strengthening, breathing exercises and sit-to-stand activities are all prescribed. Active pelvic bridging is used in conjunction with a brace to enhance core activation while walking to improve lower limb strength [17]. According to Carolus et al., necrolysis and nerve decompression are necessary for total nerve compression. For patients who have a permanent foot drop, muscle-transfer surgery may be beneficial [18]. According to Cho et al., exercises to increase pelvic floor muscle strength, power, endurance, and relaxation are called pelvic floor muscle exercises (PFME). PFME has been suggested as the first-line treatment for urine incontinence, especially stress urinary incontinence [19]. Marques et al. conducted a study on patients with stress incontinence, in which they found that throughout the sessions, the frequency of micturition was reduced when the pelvic floor muscle (PFM) and hip synergic muscles were strengthened [20].

## Conclusions

Typically, severe foot drop cases undergo surgical intervention. However, considering the minimal findings and lack of severity in the mentioned patient, a specialized physiotherapy exercise plan was implemented to enhance the lower limb strength and address the left foot drop. Integrating this physiotherapy plan with other treatment approaches resulted in improved gait patterns, balance, and performance in daily activities. The patient achieved the ability to walk with minimal assistance and expressed heightened confidence. This study highlights the efficacy of physiotherapy exercise programs in enhancing outcomes for individuals with lumbar canal stenosis.
